# Investigating the properties and antibacterial, antioxidant, and cytotoxicity activity of postbiotics derived from Lacticaseibacillus casei on various gastrointestinal pathogens in vitro and in food models

**DOI:** 10.3205/dgkh000515

**Published:** 2024-11-05

**Authors:** Zahra Asadi, Amin Abbasi, Ali Ghaemi, Effat Abbasi Montazeri, Sousan Akrami

**Affiliations:** 1 Student Research Committee, Ahvaz Jundishapur University of Medical Sciences, Ahvaz, Iran; 2 Department of Food Science and Technology, National Nutrition and Food Technology Research Institute, Faculty of Nutrition Science and Food Technology, Shahid Beheshti University of Medical Sciences, Tehran, Iran; 3 Department of Microbiology, Faculty of Medicine, Ahvaz Jundishapur University of Medical Sciences, Ahvaz, Iran; 4 Students' Scientific Research Center (SSRC), Tehran University of Medical Sciences, Tehran, Iran

**Keywords:** cell-free supernatant probiotic, Lacticaseibacillus casei, antibacterial capacity, gas chromatography/mass spectrometry

## Abstract

**Background:**

Postbiotics comprise soluble compounds freed from the structure of destroyed bacteria or created by living bacteria. Such byproducts provide the host with enhanced biological function as well as specific physiological consequences. This research aims to examine the characteristics and possible health advantages of *Lacticaseibacillus (L.) casei*-derived postbiotics.

**Methods:**

The antibacterial effects of postbiotics derived from *L. casei* were examined in vitro against various infectious gastrointestinal agents, as well as pasteurized milk and minced beef. Postbiotic activity potential was evaluated using disc-diffusion agar, minimum inhibitory concentration, minimum bactericidal concentration, and well-diffusion agar methods. Postbiotics were tested for antioxidant activity against 2,2-azinobis (3-ethylbenzothiazoline-6-sulfonic acid) (ABTS) free radicals. Additionally, the total phenolic and flavonoid content of the postbiotics was determined. The colorimetric MTT was used to investigate the potential cytotoxicity of postbiotics. The chemical makeup of the postbiotics was also determined using gas chromatography/mass spectrometry.

**Results:**

The antibacterial capacity was mostly related to pyrrolo[1,2-a] pyrazine-1,4-dione, benzoic acid, and laurostearic acid. Gram-positive microbes were more influenced by microbial byproducts in vitro than Gram-negative bacteria (P<0.05). The minimum effective concentrations of postbiotics were found to be much greater in ground beef and milk in the *Listeria monocytogenes*-inoculated model than with other bacteria (P<0.05). Postbiotics also show high antioxidant activity. Postbiotics generated from *L. casei* had the greatest concentrations of phenolic (99.46 mg GAE/g) and flavonoid (17.46 mg QE/g) constituents. Postbiotics had no influence on the viability of human foreskin fibroblasts at any dose.

**Conclusion:**

*Lactobacillus* spp. postbiotics, particularly *L. casei*, were recommended for use as antioxidants, antimicrobials, and preservatives in both the food and pharmaceuticals sector for their beneficial effects and biological properties.

## Zusammenfassung

**Hintergrund:**

Postbiotika bestehen aus löslichen Verbindungen, die aus der Struktur zerstörter Bakterien freigesetzt oder von lebenden Bakterien erzeugt werden. Diese Nebenprodukte bieten dem Wirt eine verbesserte biologische Funktion mit spezifischen physiologischen Auswirkungen. Ziel der Untersuchung war es, die Eigenschaften und möglichen gesundheitlichen Vorteile von Postbiotika aus L*actaseibacillus (L.) casei* zu untersuchen.

**Methode:**

Die antibakterielle Wirkung von Postbiotika aus *L. casei* wurde in vitro sowie in pasteurisierter Milch und Hackfleisch gegen verschiedene gastrointestinale Infektionserreger untersucht. Die Wirksamkeit der Postbiotika wurde mithilfe des Agardiffusionstests (Scheibchen- und Lochtest), der Bestimmung der minimalen Hemmkonzentration und der minimalen bakteriziden Konzentration bewertet. Die Postbiotika wurden auf antioxidative Aktivität gegen 2,2-Azinobis(3-ethylbenzothiazolin-6-sulfonsäure) (ABTS)-freie Radikale getestet. Zusätzlich wurde der Gesamtphenol- und Flavonoidgehalt der Postbiotika bestimmt. Der kolorimetrische MTT-Assay wurde verwendet, um die potenzielle Zytotoxizität von Postbiotika zu untersuchen. Die chemische Zusammensetzung der Postbiotika wurde mithilfe der Gaschromatographie-Massenspektrometrie bestimmt.

**Ergebnisse:**

Die antibakterielle Wirksamkeitt war hauptsächlich auf Pyrrolo[1,2-a]pyrazin-1,4-dion, Benzoesäure und Laurostearinsäure zurückzuführen. Grampositive Bakterien wurden in vitro stärker von den mikrobiellen Nebenprodukten beeinflusst als Gramnegative Bakterien (P<0,05). Die minimal wirksamen Konzentrationen von Postbiotika waren in Hackfleisch und Milch im mit Listeria monocytogenes inokulierten Modell viel höher als mit anderen Bakterien (P<0,05). Postbiotika zeigen auch eine hohe antioxidative Aktivität. Aus *L. casei* erzeugte Postbiotika hatten die höchsten Konzentrationen an phenolischen (99,46 mg GAE/g) und flavonoiden (17,46 mg QE/g) Bestandteilen. Postbiotika hatten in keiner Dosis Einfluss auf die Lebensfähigkeit menschlicher Vorhautfibroblasten.

**Schlussfolgerung:**

Postbiotika von *Lactobacillus* spp., insbesondere aus *L. casei*, werden aufgrund ihrer wünschenswerten Wirkungen und biologischen Eigenschaften zur Verwendung als Antioxidantien, antimikrobielle Mittel und Konservierungsmittel sowohl im Lebensmittel- als auch im Pharmasektor empfohlen.

## Introduction

Over the last few decades, researchers have paid special attention to prebiotics, probiotics, and postbiotics. They are acquired from many sources (for example, fruit, vegetables, and dairy products) and employed in a variety of foods and beverages because of their therapeutic benefits [1], e.g., enhancing the shelf life of foods. Functional foods have been created using probiotics and prebiotics, and in response to customer desire for chemical-free meals, a recent developing trend in the application of postbiotics in diverse foods has evolved. Furthermore, the use of probiotics in meals has been shown to have certain benefits [2]. Postbiotics, for instance, are more resistant to food processing and may survive for a lengthy amount of time. The use of live probiotic bacteria in comestibles has its own set of constraints. Food type, pH, temperature, and other factors have a significant impact on live probiotic bacteria. Massive quantities of prebiotics and probiotics are being used in the food industry to manufacture functional foods, extend shelf life, and improve food safety [3].

Producer strains of bacterial and fungal species *(Lactobacillus*, *Bifidobacterium*, *Streptococcus*, *Akkermansia* *muciniphila*, *Saccharomyces* *boulardii*, *Eubacterium* *hallii*, *Faecalibacterium*, etc.) from which postbiotics are recovered in situ can be found naturally in a variety of fermented foods [4]. As researchers discovered a relationship between the microbiome and immune support as well as overall health in recent years, gut health and dietary items that promote gut health have piqued the public’s attention. Postbiotics are the most recent contribution to the discussion about gut health [5]. Whenever prebiotics are consumed, gastrointestinal microbes digest them, producing beneficial postbiotics as a result [6].

*L. casei* is one such helpful bacterium that has been found to create a variety of postbiotics with a range of advantages [7]. Organic acids (such as lactic acid), bacteriocins (antimicrobial peptides), exopolysaccharides (complex sugars), and some enzymes are examples of postbiotics generated from *L. casei*. These postbiotics are expected to add to the general health-promoting benefits of *L. casei* probiotic supplements or fermented foods [8]. Postbiotics generated from *L. casei* have been proven in studies to have anti-inflammatory, immune-modulating, and antibacterial properties. They have also been shown to increase intestinal barrier integrity, lower oxidative stress, and boost nutritional absorption [9]. In general, *L. casei* postbiotics offer a viable path for the creation of novel functional foods and nutraceuticals that might improve health and promote disease prevention [10].

The *L. casei* strain has been studied less than other lactic acid bacteria. This study investigated the antibacterial, antioxidant, and cytotoxicity activities of these postbiotics on numerous gastrointestinal microorganisms in vitro and in food models. The intention was to obtain a better understanding of their potential as a natural cure for numerous digestive problems by studying the impact of these postbiotics on bacteria typically present in the human gut. Furthermore, we looked at whether these postbiotics may serve as antioxidants, which could provide further general-health advantages. Finally, we investigated the cytotoxicity of these postbiotics in order to identify any possible dangers related to their usage.

## Materials and methods

### Materials

Merck Millipore (Darmstadt, Germany) provided the yeast peptone dextrose (YPD), yeast malt broth (YMB), agar-agar, 100% ethanol, and chloroform. Sigma-Aldrich Co (St Louis, MO, USA) provided the ABTS (2,2'-azino-bis [3-ethylbenzothiazoline-6-sulfonic acid] diammonium salt), gallic acid, and quercetin. Mueller Hinton agar (MHA), Mueller Hinton broth (MHB), Sabouraud dextrose agar (SDA), mannitol salt agar (MSA), eosin methylene blue (EMB), plate count agar (PCA), brain heart infusion (BHI), Luria Bertani (LB), and cefixime tellurite sorbitol MacConkey (CT-SMAC) were all obtained from Merck Millipore.

### Preparation and maintenance of probiotic yeast strain

Persian Type Culture Collection (Iranian Research Organization for Science and Technology) supplied *L. casei* (PTCC 1608) and was cultivated in YPD (20 g/L dextrose, 4 g/L yeast extract, 3 g/L bacterial peptone, 2 g/L Tri ammonium citrate, 1 g/L polysorbate 80, 1 g/L KH2PO4, and 0.8 g/L MgSO4) for 24–48 h at 30°C while being stirred at 200 rpm. If necessary, *L. casei* suspensions were standardized using a spectrophotometer (Shimadzu UV3600, Japan) in the 600-nm (ultraviolet) wavelength range.

### Preparation of yeast cell-free supernatant solution

The aerobic cultivation of *L. casei* in YMB for 48 hours at 37°C is the initial step in the production of cell-free supernatant (CFS) postbiotics. Because varied microbial strain growth conditions and extraction methodologies have a major impact on postbiotic efficacy, this approach was carried out in 2,000-mL Erlenmeyer flasks to obtain adequate and suitable amounts of CFSs. In brief, CFS was extracted after 24–48 hours of incubation at 37°C, followed by 10 minutes of centrifugation at 4°C and 4,500 rpm. Before treating the samples, the yeast medium supernatant was collected, the pH was adjusted to 7.2, and it was filtered through a 0.22-m Millipore filter (Sigma-Aldrich, Millipore Sigma Co., Germany). The filtrate was collected in order to freeze-dry it. The extracted CFS were then frozen for 24 hours at –80°C. From 40°C to 30°C, at 0.2 mbar, the CFS was lyophilized (Lyophilization Systems, Inc, USA). The entire freeze-drying procedure took 24 hours, and the freeze-dried powders were kept at 20°C. Prior to usage, they were rehydrated with sterile, deionized water.

### Gas chromatography/mass spectroscopy (GC/MS)

By using a mass spectrometer (Agilent Technologies 5975C, USA) in conjunction with a gas chromatograph (Agilent Technologies 7890A, USA), the primary chemical constituents of postbiotics that originate from *Lacticaseibacillus casei* (PLC) were identified and quantified. A capillary column (with dimensions of 30 m×0.25 mm×0.25 µm) was injected with PLC (0.1 µL), and its temperature was raised from 45°C to 210°C at a rate of 3°C/min. The ionization energy was fixed at 70 eV, and the helium gas flow rate was 1 mL/min. 

### Assessment of the Total Phenol Content

Folin-Ciocalteu reagent was used to assess the total phenolic content of the PLC using a method established by Abeysekera et al. [11]. The Folin-Ciocalteu reagent (110 µL) and sodium carbonate solution (70 µL) were used to charge the PLC (20 µL). After 30 minutes at 25°C, the mixture’s absorbance was measured at 765 nm. Gallic acid (0.06–1 mg/mL) was used to create the standard curve, and the total phenol content of PLC was determined as mg gallic acid equivalent (GAE)/g PLC.

### Assessment of the total flavonoid content

The total flavonoid content of PLC was assessed using the aluminum chloride method. The mixture of PLC (100 µL) and aluminium chloride (100 µL; 2% w/v in methanol) was incubated at 25°C for 10 minutes before the absorbance at 367 nm was measured. The calibration curve was created using quercetin (7.81–125 mg/mL), and the total flavonoid content was reported as mg quercetin equivalent (QE)/g PLC [11].

### Antioxidant activity

PLC’s antioxidant capacity was evaluated in relation to ABTS free radicals. The oxidation of 2.45 mM K_2_S_2_O_8_ with a 7 mM ABTS solution produced the ABTS radical monocation. The combination was kept at room temperature for 12 hours in a dark environment before being diluted with ethanol to achieve 0.70.2 absorbance at 750 nm. The ABTS solution (1.8 mL) was then mixed with the PLC or control (0.2 mL), and the mixture’s absorbance at 750 nm was measured using a spectrophotometer. Following is a calculation of CZEO’s antioxidant activity (Equation 1):


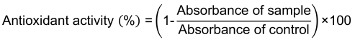


### Antibacterial activity assessment of the prepared postbiotics in vitro

The antibacterial effectiveness of the postbiotics was evaluated using the disc-diffusion agar (DDA) method, minimum inhibitory concentration (MIC), minimum bactericidal concentration (MBC), and well-diffusion agar (WDA) against *Salmonella* *typhi* ATCC 6539, *Escherichia* *coli* ATCC 25922, *Streptococcus* *mutans* ATCC 25175, *Clostridium* *difficile* ATCC 9689, and *Listeria* *monocytogenes* ATCC 19112. The antibacterial properties of tetracycline, gentamicin, and chloramphenicol were compared in the DDA test.

### Antibacterial activity assessment of the prepared postbiotics in food models

According to Hartmann et al. [12], the minimum effective concentration (MEC) of postbiotics was determined in the same way. *E*. *coli* ATCC 25922 and *L*. *monocytogenes* ATCC19112 were added to 10 mL of pasteurized milk in a bottle and 100 g of ground meat in sterile wrappers, respectively, to bring the final population to an appropriate level (~3.2 and 4.1 (*E. coli*), 3.5 and 4.6 (*L. monocytogenes*) lg colony forming units (CFU)/mL or gram of milk and meat, respectively). PLC was added to the milk and meat samples at concentrations ranging from 10 to 60 mg/mL, and each sample was completely homogeneous. All samples were kept at 4°C for six days. Utilizing PALCAM Listeria Selective Agar and Cefixime Tellurite Sorbitol MacConkey agar, the studied pathogens were cultivated and counted. MEC was defined as the PLC concentration that decreases the initial microbial population to below the culture limit of 10 bacteria for milk and 100 bacteria for meat over the course of three days at 4°C. Instead of a food matrix, comparable experiments were conducted using BHI broth and LB broth. Assays were performed in triplicate.

### Cytotoxicity of the postbiotics

Human foreskin fibroblasts (HFF) were used to evaluate the possible toxicity of postbiotics of *L*. *casei* using the colorimetric MTT [3-(4, 5-dimethylthiazol-2-yl)-2, 5-diphenyltetrazolium bromide] test. HFF cells were exposed to PLC at various doses for the predetermined amount of time (24 h) (1x10^5^ cells/0.3 mL DMEM cell culture medium in 96-well plates). After 24 hours, the medium was withdrawn, and MTT solution was added to the cells in each microplate (0.2 mL; 3 mg/mL). The MTT solution was removed after 3 hours of incubation at 37°C, and formazan was then solubilized by adding 100 µL/well of lysis buffer. Using a microplate reader (Bio-Rad Model 3550), optical density (OD) was assessed at 570 nm after the plate had been shaken. The percentage of untreated (controls) cells was used to express cell viability as follows (Equation 2):


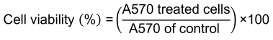


### Statistical analysis

All data were analyzed using GraphPad Prism (GraphPadSoftware; San Diego, California, USA). Levene’s test was carried out to ensure the equality of variances. Results are shown as mean± SD (n=3). The threshold for significance was set at P<0.05.

## Results

### Chemical composition of postbiotics

Table 1 (Tab. 1) displays the chemical profiles, retention times, and major constituent percentages (>1%) of postbiotics from *L. casei*. Organic acids, peptides, substances comprising fatty acids, alcohols, esters, and aldehydes in various forms and concentrations were abundant in postbiotics. The ipostbiotics examined were found to contain pyrrolo[1,2-a] pyrazine-1,4-dione, benzoic acid, and 5,10-diethoxy-2,3,7,8-tetrahydro-1H,6H-dipyrrolo[1,2-a:1',2'-d]pyrazine. These substances are well-known for their antibacterial and antioxidant properties [13]. Among the substances detected, postbiotic metabolites contained laurostearic acid, a waxy, 18-carbon-chain, saturated fatty acid. Laurostearic acid may function as a biosurfactant and aid in clearing the surface of the pathogen biofilm [14]. Additionally, the presence of 1, 4-diaza-2, 5-dioxo-3-isobutyl bicyclo[4.3.0]nonane among the detected chemicals may point to the generated postbiotic solution’s possible anti-biofilm and antimicrobial effect [15]. Another substance with promising antibacterial and anti-inflammatory characteristics is ergotaman-3',6',18-trione, 12'-hydroxy-2'-methyl-5'-(phenylmethyl)-, (5'alpha) [16]. One of the characteristics of lactic acid bacteria is the formation of organic acids, such as lactic acid and acetic acid. Although certain organic acids were present, lactic acid was not among the postbiotic metabolites that were analysed. The findings of the experiments demonstrated that the type and composition of individual chemicals are significantly influenced by the technique used for analysis.

### Total phenols and flavonoids of the prepared postbiotics

The PLC contains significant amounts of flavonoids (17.46 mg QE/g) and phenolics (99.46±0.12 mg GAE/g) (P<0.05). Indeed, growth and extraction conditions, coupled with basic stock preparation techniques, have an impact on the content and quality of the final produced PLC solution. The studied PLC had significant amounts of phenolic and flavonoid compounds when all of the active components were taken into account.

### Antioxidant activity

Due to the fact that polyphenols contain natural redox capabilities that can neutralize free radicals, these bioactive substances have the ability to strengthen the immune system while also providing preventative benefits for a number of degenerative illnesses, including diabetes and cardiovascular disease [17]. The direct oxidation of ABTS with K_2_S_2_O_8_ results in a stable, blue-green ABTS●+, which is the basis for the ABTS test. The amount of the radical solution’s decolorization (reduction) is then measured at 750 nm when antioxidants are present [18]. The PLC’s antioxidant activity was quite high (70.41+0.43%). Phenols, flavonoids, and the primary chemical components of the PLC all contribute to this exceptionally strong antioxidant action [19]. Some research has demonstrated the PLC’s antioxidant ability. As a result, the PLC might be employed to slow down or stop the oxidative damaging effects of free radical processes. 

### Antibacterial activity of the prepared postbiotics in vitro

This study used antimicrobial tests, including DDA, WDA, MIC, and MBC against harmful bacterial species to assess the PLC’s antibacterial potential. The antibacterial action of the PLC varied depending on the yeast types, and Gram-positive bacteria were more commonly impacted by microbial byproducts than were Gram-negative bacteria (Table 2 (Tab. 2) and Table 3 (Tab. 3)). In the DDA and WDA tests, the mean inhibition zones for Gram-positive bacteria were 19.61 mm and 23.24 mm, respectively, whereas the mean inhibition zones for Gram-negative bacteria were 10.90 mm and 15.16 mm, respectively. The MIC and MBC experiments produced similar results, and less PLC was needed to kill or stop the growth of Gram-positive bacteria than Gram-negative bacteria (Table 3 (Tab. 3)).

### Antibacterial activity of the prepared postbiotics in food models

The amount of an antimicrobial agent that must be present for it to be effective at preventing the growth of a pathogen in a food product is known as the minimum effective concentration (MEC). The estimated MECs of PLC against *L. monocytogenes* and *E. coli* in various challenge matrices are shown in Table 4 (Tab. 4). In the *L. monocytogenes* inoculation model, the MECs of PLC were much higher in milk and ground beef, although the MECs varied greatly among the different dietary models. When exposed to an *E. coli*-infected model, PLC demonstrated little antibacterial activity (50 mg/mL in food models), which is comparable to *in vitro* settings. The influence of structural variations between Gram-positive and Gram-negative bacteria on the antibacterial capabilities of PLC was brought to light by this. 

### Cytotoxicity of postbiotics

PLC did not show any cytotoxic effect at any concentration on the viability of HFF. Interestingly, PLC simulated the growth of HFF cells at high concentrations (100 and 1,000 µg/mL). Additionally, a dose-dependent cytotoxic effect has been reported on myeloma cells proliferation for the suspensions of L. salivarius HA8 and Enterococcus faecium CH3 [20]. 

## Discussion

Bioactive chemicals such as probiotics, prebiotics, and postbiotics have received much attention in recent decades. These substances are associated with the beneficial gut microbiota and promote host health. Postbiotics are live probiotic components that can execute the biological and physiological tasks of their natural parent organisms [21]. Postbiotics often exhibit their health and therapeutic benefits via modes of action that are comparable to or distinct from those of their predecessors. Postbiotics are less susceptible to processing circumstances, environmental influences, and gastrointestinal conditions than probiotics, which increases their commercial viability [22]. *Salmonella typhi*, *Escherichia coli*, *Streptococcus mutans*, *Clostridioidis (C.) difficile*, and *Listeria monocytogenes* are known to cause foodborne illnesses in humans. These microorganisms can infect food due to a variety of factors, including poor sanitation practices during the preparation of food, insufficient cooking, and incorrect storage conditions. *S. typhi* produces typhoid fever, a kind of food poisoning marked by symptoms including high fever, stomach discomfort, and diarrhea [23]. *E. co*li is a prevalent source of food-related illness that can cause stomach discomfort, diarrhea, and nausea. *S. mutans* is frequently linked to dental caries [24]. *C. difficile* has been linked to major diarrhea and colitis, while *Listeria monocytogenes* can cause listeriosis, a potentially fatal infection of the brain and spine [25]. Because its environment becomes acidic during its growth, the antibacterial activity of *L. casei* supernatant can be related to the generation of organic acids: pathogenic organisms which are susceptible to acidic environments are destroyed [26]. Many infections have been demonstrated to cause malignancy in humans [27]. Salmonella and gallbladder cancer have been linked in studies. The host’s health is substantially influenced by the gut flora [28]. For instance, a heat-killed *L. delbrueckii*-supplemented diet stimulates the establishment of desirable *Bifidobacterium* spp. and related metabolic alterations in the individual's fermented fecal population [29]. Short-chain fatty acids maintain the pH of the human stomach at 5-6, limiting detrimental bacteria while enabling health-promoting bacteria to grow [30]. According to Kotani et al.,* L. rhamnosus* inhibited the dehydration and oxidation of bile acids by lowering the activity of β-glucuronidase, hence suppressing microbial enzymes [31]. Furthermore, *L. plantarum* b240 inhibited the invasion and adhesion of bacterial pathogens onto intestinal epithelial cell surfaces. A postbiotic-rich diet has the ability to change the substance, variety, and richness of the gut microbiota [31]. This study performed antimicrobial assays such as DDA, WDA, MIC, and MBC against spoilage and dangerous bacterial species to evaluate the PLC’s antibacterial properties. Based on the findings of the DDA and WDA tests, *L. monocytogenes* and *E. coli* exhibited the largest and smallest inhibition zones, respectively, for the antibacterial activity of the PLC. Furthermore, the inhibitory zone resulting from the WDA technique was much larger than that of the DDA test. This is partly due to the fact that the former technique directly interacts with the bacterial species, while the latter does not [32]. A synergistic effect of PLC and antibiotics is also worth noting; this combination exhibited atypically better antibacterial effects than the PLC alone (Table 2 (Tab. 2)). In this investigation, Gram-positive bacteria required less PLC to be killed or stop growing than did Gram-negative bacteria. Based on the MIC and MBC data, these differences have been linked to variances in the bacterial cell wall structure. Lipoproteins and lipopolysaccharides are major components of Gram-negative bacterial walls, which contribute to their superior resistance to antimicrobial agents [18].

High amounts of reactive oxygen species and free radicals expose biological systems to oxidative stress. The existence of oxidative stress has been linked to a number of disorders, including cancer, cirrhosis of the liver, and fatty liver [33]. Exopolysaccharides (EPSs) from lactic acid bacteria (LAB) were found to help eliminate free radicals, functioning as natural powerful antioxidants. They have also been shown to be non-toxic, implying that they might be utilized as a substitute for synthetic antioxidants, which pose certain hazards [34]? In the current investigation, PLC has a significantly higher antioxidant activity (70.41±0.43%). In-vitro and *in vivo* research have extensively explored the good antioxidant activity of LAB-derived EPSs. Dilna et al. [33] isolated EPS from L. plantarum RJF4, which comprised glucose and mannose. EPS boosted overall antioxidant effectiveness by 32%, radical scavenging efficiency by 24%, and reduction potential by 50% (when compared to ascorbic acid as a benchmark). EPS from *L. acidophilus* demonstrated strong in-vitro antioxidative action on lines of colon cancer cells in DPPH (2,2-diphenyl-1-picrylhydrazyl) -radical scavenging, reducing power, and ferric reducing antioxidant power (FRAP) tests. In all of these experiments, raising the concentration of EPS increased its antioxidative properties [35].

In recent years, functional foods including probiotics, prebiotics, and postbiotics have attracted increasing interest of researchers, producers, and consumers. There is currently a large range of bioactive food items on the market, such as probiotics, dairy, and non-dairy products, to meet the nutritional demands of consumers with varied dietary needs and choices, such as those who are allergic to milk proteins, lactose intolerant, and vegetarians. Because postbiotics are stable throughout a wide temperature and pH range, they can be added before thermal processing without impairing their functionality. This may provide producers with certain technical and economic benefits [36]. In the present study, the MECs of PLC were shown to be considerably greater in ground beef and milk in the *L. monocytogenes* inoculation model. According to Ahmad Rather et al., the postbiotic supernatant of *L. plantarum* YML007 can be used as a bio-preservative, prolonging the shelf life of soybeans by up to two months [37]. *L. lactis* subsp. *lactis* may generate nisin, which is used as a preservative in a variety of foods (infant formula, canned soups, and dairy products) [38]. With the foregoing in mind, the use of foods as a delivery mechanism for postbiotics appears to be a promising but challenging endeavor. 

PLC had no cytotoxic effect on the viability of HFF at any concentration. Furthermore, *Lacticaseibacillus* suspensions have been shown to have a cytotoxic effect on cancer cells. Cancer therapies are recognised to have limits and adverse effects, prompting researchers to look for alternate therapy options for generating innovative, safe and effective CRC remedies. Probiotics, prebiotics, synbiotics, paraprobiotics, and metabiotics or postbiotics are the most well-investigated options in this context [39]. Elham et al. [40] accumulated substantial data confirming the anti-colon cancer properties of *L. casei*. A dose-dependent cytotoxic impact was reported, with increasing concentrations of the fractions causing increased cytotoxicity in Caco-2 cells. Furthermore, several LAB strains have been discovered to have dose-dependent anticarcinogenic properties [41]. Live probiotic cells have an impact on both the gastrointestinal microbiota and the immune response, whereas heat-killed cells have an anti-inflammatory effect on the gastrointestinal tract. *Lacticaseibacillus* spp. cell wall components are known to elicit an inflammatory response involving macrophages in the mammalian gastrointestinal tract [40]. Another group of researchers [42] reported that live and HK-sonic protein of *L. casei* induced cytotoxicity effects in vitro on CT-26 and HT-29 cells and were able to reduce the viability of murine CT-26 (colon cancer cell line) and human HT-29 (colon cancer cell line).

GC/MS was used to identify and quantify the chemical makeup of PLC. Postbiotics are bioactive chemicals created following the development and metabolism of probiotic bacteria such as *L. casei*. The chemical makeup of postbiotics varies based on the *L. casei* strain, fermentation circumstances, and growth medium employed. Organic acids, bacteriocins (antimicrobial peptides), EPS, phenolic compounds, and enzymes are some of the most prevalent bioactive molecules observed in *L. casei* postbiotics. Several studies have reported on the chemical makeup of *L. casei* postbiotics generated under diverse settings [8]. Overall, the chemical composition of postbiotics from *L. casei* is diverse and complex, and further research is needed to fully understand its potential health benefits.

## Conclusions

*Lacticaseibacillus* products and by-products have been discovered to have several therapeutic roles, including epithelial barrier maintenance, anti-tumor impact, immunomodulation, and antagonistic actions against infections. Furthermore, they offer some benefits over probiotics, such as clear chemical structures, safety dosing guidelines, and a longer shelf life. Postbiotics are increasingly being used in the human food, animal feed, and pharmaceutical sectors, and various paraprobiotic and postbiotic products generated from *Lactobacill* species are commercially accessible for disease prevention or treatment. Nonetheless, further data are required to establish postbiotics’ positive benefits. According to the findings of this study, postbiotic metabolites derived from *Lacticaseibacillus casei* (PTCC 1608) have multiple health-promoting effects due to their significant antioxidant capacity (P<0.05) and antibacterial action against both Gram-negative and Gram-positive bacteria. Also, they can cause significant cytotoxicity (P<0.05) against and arrest the cell cycle of cancer cells. Therefore, would make sense to construct particular mixes of postbiotics with substantial efficiency by learning more about lactic acid bacteria, employing established and refined procedures for extraction, verification, characterization, and conducting metabolomic and proteomic research. This would significantly improve the performance of health-care systems in dealing with a wide range of acute and chronic disorders. Further research into postbiotics will generate fresh insights and various favorable impacts, allowing them to be used more widely in the food and pharmaceutical sectors. More research should be done to determine the factors, such as eating habits that may influence the use of these beneficial chemicals in functional foods. These initiatives will contribute to the manufacture of safe, natural, clean label goods that are also free of unwanted by-products, thus safeguarding human health. Essentially, a clean label means making a product using as few ingredients as possible and ensuring those ingredients are items that consumers recognize and think of as wholesome – ingredients that consumers might use at home.

Overall, we feel that this study provides useful information on the possible benefits and drawbacks of employing *L. casei* postbiotics to promote digestion. We intend to pave the road for the creation of innovative and efficient natural therapies for diverse digestive illnesses by better understanding the characteristics and potential health advantages of these postbiotics.

## Notes

### Author’s ORCID

Sousan Akrami: 0000-0001-6643-140X

### Ethical approval 

Protocols for collection of samples as well as the experimental design and all methods were performed in accordance with the guidelines and regulations of Ahvaz Jundishapur University of Medical Sciences and approved by the institutional ethical committees. The present research was supported by the Ahvaz Jundishapur University of Medical Sciences, Ahvaz, Iran (01s56) (No. IR.AJUMS.REC.1401.428).

### Funding 

This work was supported by grant number 01s56 from Vice Chancellor for Research, Ahvaz Jundishapur University of Medical Sciences, Student Research Committee, Ahvaz Jundishapur University of Medical Sciences, Ahvaz, Iran.

### Acknowledgments 

The present research was supported by the Ahvaz Jundishapur University of Medical Sciences, Ahvaz, Iran (01s56) (No. IR.AJUMS.REC.1401.428).

## Figures and Tables

**Table 1:**
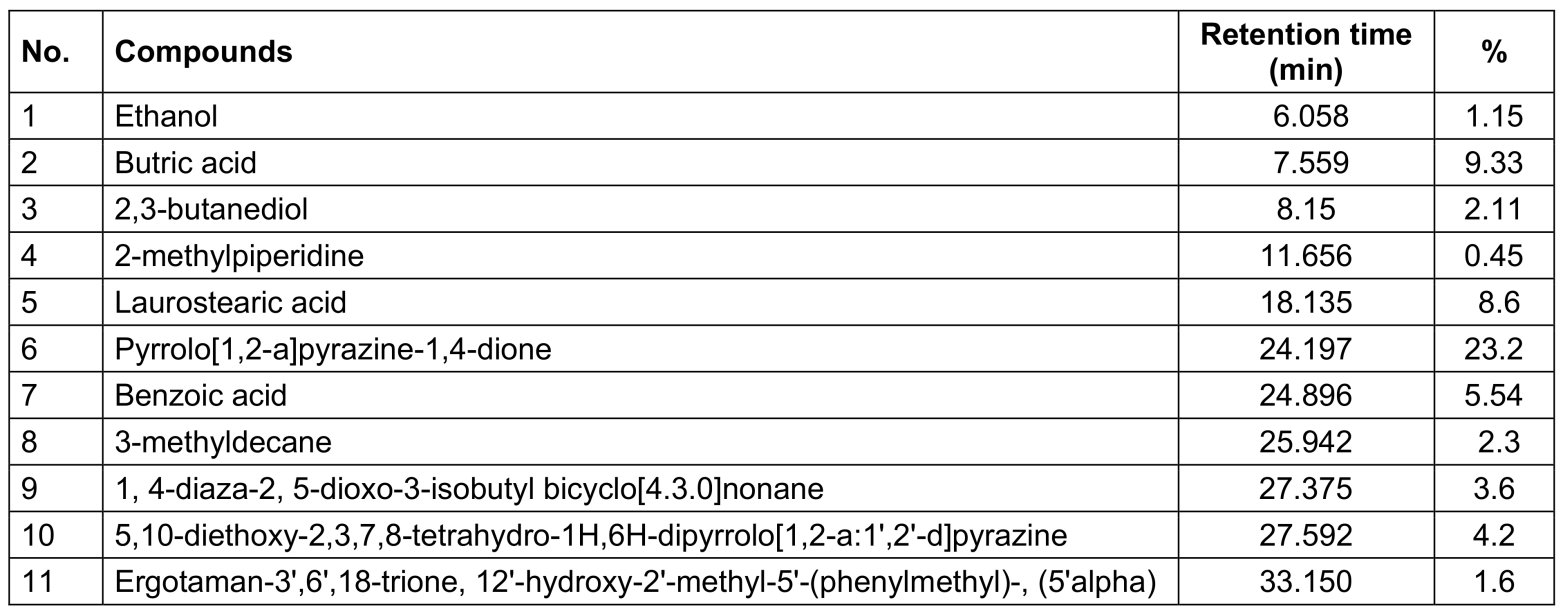
Chemical composition of PLC identified and quantified by GC/MS

**Table 2:**
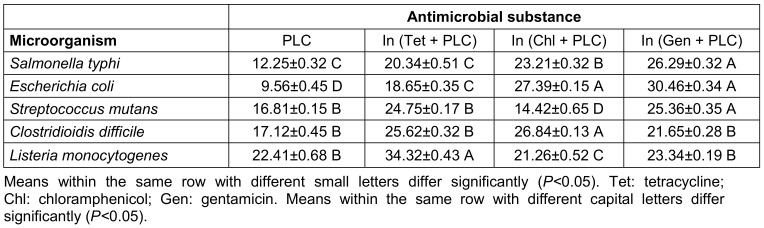
The mean inhibition zone diameter (mm) of PLC for some pathogenic microorganisms by disc diffusion agar (DDA) method

**Table 3:**
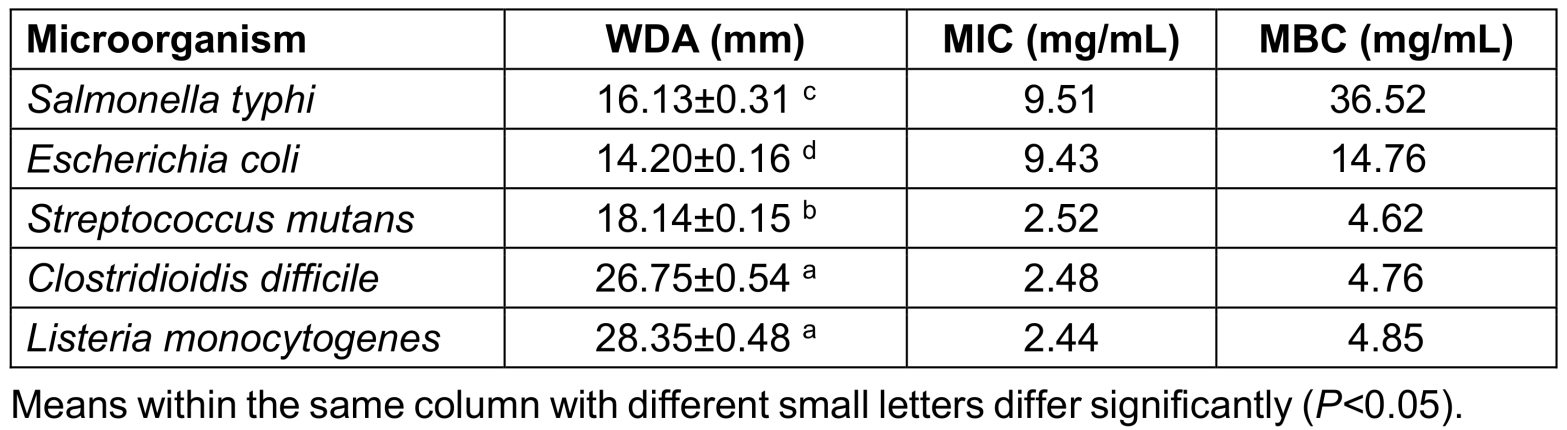
The well-diffusion agar (WDA), minimum inhibitory concentration (MIC) and minimum bactericidal concentration (MBC) of the PLC on some pathogenic microorganisms

**Table 4:**
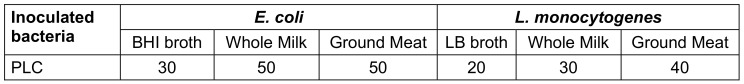
Minimal effective concentration (MEC) (mg/mL) of the PLC on *E. coli* and *L. monocytogenes* in whole milk, ground meat and culture media
